# Supplementary material for: A high-resolution mRNA expression time course of embryonic development in zebrafish
Source: eLife. 2017 Nov 16;6:e30860. doi: 10.7554/eLife.30860 (PMC5690287; doi:10.7554/eLife.30860)
Supplement: Supplementary file 6. [file elife-30860-supp6.zip › biolayout-clusters-files/Cluster192-genes.html]

Cluster192


# Cluster192: Genes

| Ensembl ID | Gene Name | Chr | Start | End | Biotype |
| --- | --- | --- | --- | --- | --- |
| ENSDARG00000003984 | LTN1 | 10 | 23130432 | 23169455 | protein\_coding |
| ENSDARG00000074985 | aurkaip1 | 23 | 29956675 | 29960937 | protein\_coding |
| ENSDARG00000053097 | hsf2 | 20 | 40578466 | 40590174 | protein\_coding |
| ENSDARG00000078784 | mgaa | 17 | 10410456 | 10434037 | protein\_coding |
| ENSDARG00000063182 | rc3h1b | 2 | 7277982 | 7317988 | protein\_coding |
| ENSDARG00000044567 | ttc1 | 21 | 29970871 | 29982931 | protein\_coding |
| ENSDARG00000077581 | zzz3 | 2 | 8981056 | 9042891 | protein\_coding |
